# Supplementary material for: Preparation and Characterization of Silica-Based Ionogel Electrolytes and Their Application in Solid-State Lithium Batteries
Source: Polymers (Basel). 2023 Aug 22;15(17):3505. doi: 10.3390/polym15173505 (PMC10489929; doi:10.3390/polym15173505)
Supplement: Supplementary file 1 [file polymers-15-03505-s001.zip › polymers-2507801-supplementary.pdf]

Table S1. Cyclic discharging and charging test (discharge capacity) of CR2032 coin cell assembled with ionogels electrolyte.

**Sample code**	**Cycle 2 (mAh/g)**	**Cycle 5 (mAh/g)**	**Cycle 10 (mAh/g)**
**SIEM2**	108.7	77.1	43.5
**SIEM4**	95.6	65.0	32.5
**SIEM6**	76.5	54.5	25.2
